# Sample multiplexing in CyTOF: Path to optimize single-cell proteomic profiling

**DOI:** 10.46439/signaling.2.041

**Published:** 2024

**Authors:** Muharrem Muftuoglu, Michael Andreeff

**Affiliations:** 1 Section of Molecular Hematology and Therapy, Department of Leukemia, The University of Texas MD Anderson Cancer Center, Houston, TX, 77030, USA

## Abstract

Sample multiplexing significantly enhanced the depth of single-cell proteomic analysis in CyTOF (Cytometry by Time-Of-Flight). New polymer-based chelators have broadened the utility of metal isotopes, enabling improved tagging and simultaneous analysis of multiple samples. These approaches minimize batch effects, streamline experiments, conserve valuable samples, reduce costs, enhance throughput, and increase the accuracy of biological data, thereby facilitating novel discoveries.

## Background

In biomedical research, achieving single-cell resolution in investigation of cellular systems is of paramount importance. Ongoing research efforts to explore and decode the heterogeneity in cellular phenotype, functionality, genomics, epigenetics, and transcriptomics in biological systems have propelled significant technological advancements through innovative breakthroughs [1–3]. Among these advancements, mass cytometry, commonly referred to as CyTOF, was developed as a highly multiplexed platform for single-cell proteomic analysis [4,5]. Since its introduction over a decade ago, CyTOF has dramatically transformed single-cell proteomic analysis, expanding our capability for detailed examination and understanding of cellular heterogeneity and function.

CyTOF provides key advantages over traditional flow cytometry by enabling highly multiplexed single-cell proteomic analyses without compensation for signal spillover, effectively addressing spectral overlap issues and the capability to measure a broad array of distinct cellular features [6,7]. Although theoretically capable of detecting up to 135 unique metal isotopes, practical applications usually involve around 60 distinct features, limited by the availability of metal isotope tags and the complexity of conjugation chemistry [8–10]. This gap has sparked increasing interest in expanding the measurable parameters by incorporating additional metal isotopes, aiming to fully utilize its capacity for more detailed single-cell analysis [11–13]. Additionally, the standardization and uniformity of antibody conjugation methods facilitate the seamless incorporation of specific antibodies into the analysis panel, enabling the comprehensive interrogation of a wide range of cellular features [14,15]. CyTOF antibody conjugation is notably more streamlined, permitting the attachment of a broad spectrum of metal isotopes via a consistent methodology. Essentially, metal isotopes are loaded to a chelator, which is subsequently linked to the antibody after introduction of functional groups into the antibody structure. For instance, the maleimide-DTPA (diethylene triamine pentaacetic acid)—the most prevalent polymeric chelator used for antibody conjugation—captures all lanthanide isotopes (excluding lanthanum) as well as additional trivalent metals such as indium and bismuth [9]. Once functional thiol groups are exposed on the antibody maleimide group of maleimide-DTPA reacts with these thiol groups. This uniform approach allows for the generation and utilization of around 40 metal-tagged antibodies using a single methodology. In contrast, constructing large panels for flow cytometry presents more complexities since different fluorophores require distinct conjugation chemistry and approach [16]. Moreover, the lack of commercially available kits for various fluorophores further limits its utility in experimental setups.

The methodological superiority of CyTOF, underscored by the flexibility in panel design and antibody conjugation, highly multiplexed analysis, minimal signal spill-over, the availability of optimized sample multiplexing approaches, lack of autofluorescence, and durability and stability of metal isotopes loaded to chelators under harsh permeabilization conditions, has established it as a critical tool in high-throughput single-cell proteomic analysis across various biomedical fields [9,17]. It has been instrumental in advancing research in oncology, immunology, infectious diseases, autoimmunity, neuroscience, stem cell research, developmental biology, and drug discovery by enabling detailed analysis of cellular populations through multiplexed and high-throughput analysis, uncovering intricate cellular associations and driving discoveries [7,18–20]. Beyond the application of metal-tagged antibodies, the integration of probes equipped with unique metal reporters has further expanded the capabilities of CyTOF, enabling detailed analyses of complex cellular functions. This includes assessing antigen-specific immune responses using tetramers, the detection of hypoxia, tracking of nanoparticle distribution, cell division, measurement of protein synthesis, and monitoring of proteolytic enzyme activities [21–28]. These advancements underscore the extensive utility of CyTOF in probing complex biological systems and enabling in-depth exploration of intricate cellular dynamics, functions, and interactions.

## Sample Multiplexing in CyTOF

A key advantage of CyTOF technology is the sample multiplexing capability, allowing for the concurrent analysis of individual samples through various strategies. These include the use of metal-conjugated antibodies, chelators preloaded with metals that bind to specific targets, and specific metal compounds such as thiol-reactive tellurium (TeMal), cisplatin, and osmium (Os) and ruthenium (Ru) tetroxide [15,29–32]. These methods can be customized and combined to meet particular research needs, streamlining workflows and enhancing the scientific accuracy of cellular analysis by improving data quality, reducing variability, and enabling high-throughput analysis of numerous sample types.

In CyTOF, lanthanides primarily serve as the metal group for antibody conjugation to assess cellular features of interest [4,8,14]. Metal isotopes selected for barcoding are carefully chosen to avoid interference with those used for feature assessment, thus ensuring a seamless workflow. A significant advantage of CyTOF barcoding is the ability to use combinatorial barcoding schemes. These schemes allow a single sample to be tagged with various metals, using a limited number of metals to create a large number of unique Mass Cell Barcoding (MCB) combinations. For example, ‘6-choose-3’ or ‘7-choose-3’ barcoding schemes can produce 20 or 35 unique MCBs, respectively. This capability greatly enhances the capability for high throughput multiplexing in cellular analysis, facilitating more comprehensive and efficient data acquisition and analysis. CyTOF barcoding is typically divided into intracellular and live-cell (surface) barcoding (Table 1).

## Intracellular Barcoding

**Figure 1.**
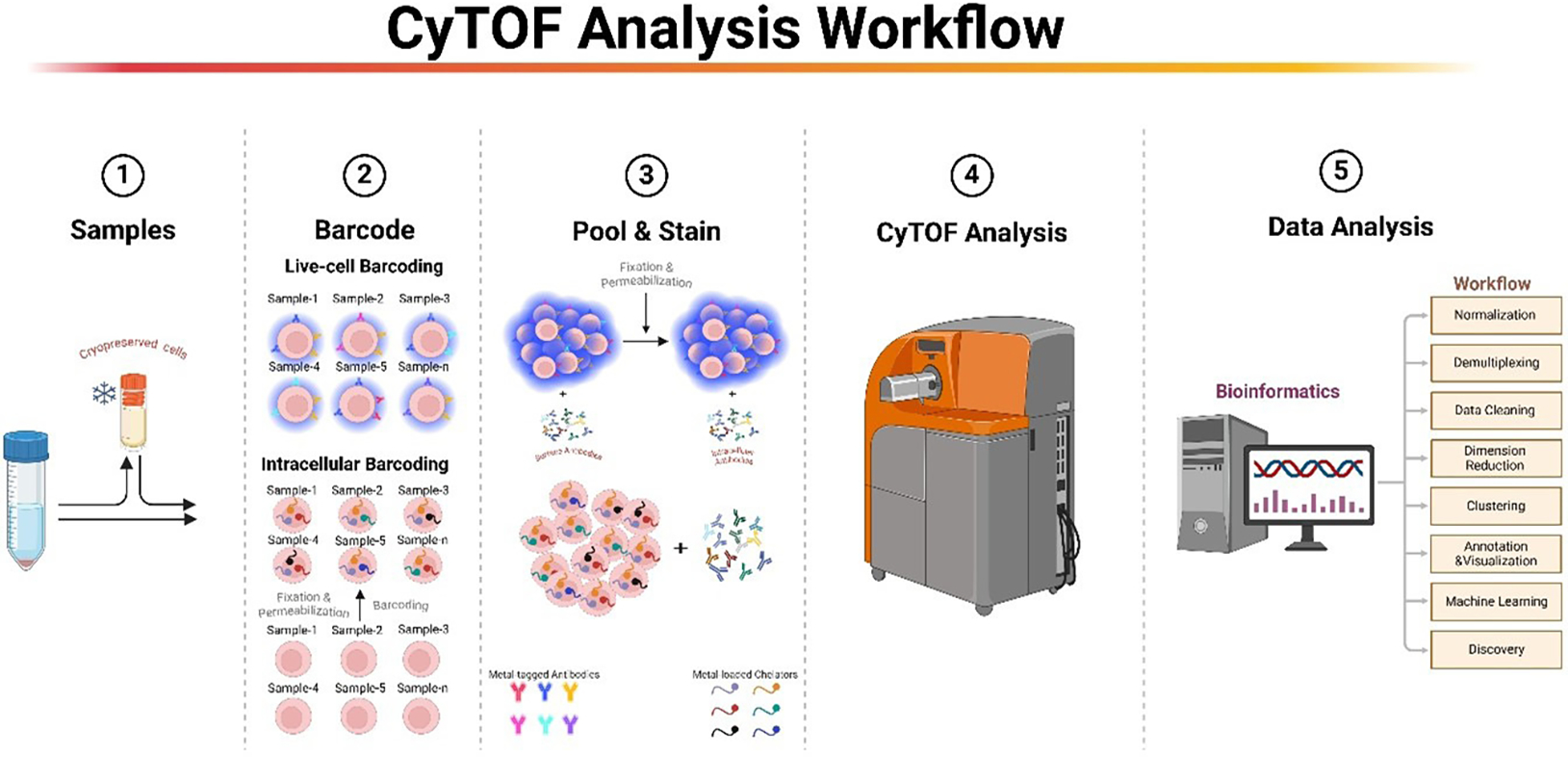
Overview of CyTOF Analysis and Sample Multiplexing Workflow. This figure illustrates the sequential steps in CyTOF analysis, starting with the preparation of single-cell suspension samples. 1) Single-cell suspensions are prepared from fresh or frozen cells. 2) For live-cell barcoding, cells are barcoded while viable using MCBs. For intracellular barcoding, cells are fixed, permeabilized, and labeled with MCBs that primarily mark intracellular targets. 3) Barcoded cells are then pooled and stained with antibodies targeting surface and intracellular molecules. 4) The pooled and stained samples undergo analysis through the CyTOF machine, which is followed by a comprehensive bioinformatics (5) analysis to interpret the high-dimensional data (Created with BioRender.com).

Intracellular barcoding in CyTOF is a methodological approach that labels cells with unique barcodes after they have undergone fixation and permeabilization [7,33] (Figure 1). This process allows barcoding reagents to effectively access and mark various intracellular targets effectively. Originating from multiplexing techniques initially developed for flow cytometry [34], this approach employs covalent binding to amine groups on cellular proteins, a principle shared by both methodologies. However, sample multiplexing techniques in CyTOF have seen wider adoption compared to flow cytometry, where the implementation of sample barcoding in flow cytometry has encountered several challenges. Flow cytometry often suffers from signal spillover between barcoding and analyte-specific channels, necessitating meticulous panel design and sometimes leading to the exclusion of certain fluorophores, which compromises the simultaneous assessment of multiple analytes [35,36]. Additionally, the allocation of a specific number of fluorophores for barcoding inherently reduces the number of available channels for analytic purposes in conventional flow cytometry. This limitation is particularly important in studies requiring the concomitant assessment of numerous cellular markers. In contrast, CyTOF provides a versatile and extensive range of parameters with minimal signal spillover and utilizes strategic metal isotope allocation that enhances cellular feature assessment without interference between barcoding and analytes.

### Advances in intracellular barcoding approach

The pioneering intracellular barcoding strategy developed by Zunder *et al.* for CyTOF employed a “6-choose-3” combinatorial scheme using Pd isotopes for MCBs [33]. This method involved six specific Pd isotopes (102Pd, 104Pd, 105Pd, 106Pd, 108Pd and 110Pd) with each barcode comprising three distinct Pd isotopes to produce 20 unique MCBs. A significant innovation in this approach is the use of a chelator, isothiocyanobenzyl-EDTA (ITCBE), which captures bivalent Pd isotopes. These barcodes are generated using monoisotopic Pd-loaded, bi-functional chelators ITCBE, which contains amine-reactive moieties, facilitating the covalent binding of the barcodes to cellular proteins. This advancement extends the use of traditional trivalent metal isotopes in CyTOF, broadening the spectrum of usable metals and enhancing sample multiplexing capabilities for high-throughput single-cell proteomic analysis. Similar to this foundational approach, subsequent strategies for intracellular barcoding have been developed. Intracellular barcoding employs various bifunctional chelators, including thiol-reactive bromoacetamidobenzyl-EDTA (BABE) [37] and thiol-reactive maleimido-mono-amido-DOTA (mDOTA) [6]. These chelators are adept at capturing metals and binding to intracellular proteins, similar to ITCBE [33]. Bifunctional chelators (mDOTA, BABE, and ITCBE) contain one functional group for creating a stable bond to the antibody and another group that captures metal isotopes, enabling precise cell labeling for CyTOF barcoding applications [15,33,37,38]. Furthermore, the integration of osmium and ruthenium tetroxides (OsO_4_ and RuO_4_) provides a unique method by leveraging their ability to form covalent bonds with fatty acids in cellular membranes and aromatic amino acids in proteins, thereby expanding the toolkit available for sample barcoding [30]. The addition of TeMal and cisplatin has also enriched these barcoding approaches [29,32]. Notably, OsO_4_, RuO_4_, TeMal, and cisplatin are versatile for use in both intracellular and live-cell barcoding applications. However, the use of these compounds requires meticulous consideration due to their propensity to influence cellular behavior, a factor that is particularly critical in live-cell barcoding scenarios.

### Challenges

A primary challenge in intracellular barcoding involves the need to fix and permeabilize cells before barcoding, which precedes both surface and intracellular staining. The fixation and permeabilization process can cause conformational changes in surface proteins, irreversibly altering antigenic epitopes that antibodies recognize [39]. These conformational changes can alter the tertiary structure of antigens which could lead to loss or masking of epitopes recognized by antibodies. This results in reduced or loss of antibody binding used for the detection of surface antigens which compromises the accurate cell surface phenotyping in conjunction with intracellular barcoding [40]. Thus, the fixation and permeabilization conditions required for intracellular barcoding are not compatible with all antibodies. This limitation can restrict the range of detectable antigens, necessitating extensive validation of antibody panels. These challenges can be mitigated by performing surface staining prior to the fixation and permeabilization steps required for intracellular barcoding [40]. Furthermore, it is important to note that some antigens, typically localized within intracellular granules, may also be affected by these procedures. Some antigens are periodically internalized into intracellular compartments before being recycled back to the surface or degraded [41]. This dynamic process can skew the accurate estimation of surface antigen expression, leading to potential misidentification of intracellular molecules as being surface expressed, especially if the staining occurs post-permeabilization. These challenges highlight the critical need for careful method selection and protocol optimization to ensure precise results in studies employing intracellular barcoding techniques.

### Conclusion

In conclusion, intracellular barcoding is crucial for high-throughput single-cell proteomic analysis in CyTOF, offering a more precise assessment of cellular behavior. Despite its associated challenges, this method **is highly effective** at accurately exploring and investigating intracellular pathways and molecular interactions, facilitating groundbreaking discoveries. Its precise application greatly improves the accuracy of single-cell proteomic analysis, enriching our understanding of cellular functions and disease mechanisms. Thus, intracellular barcoding is invaluable in advancing biological research and contributing to scientific progress.

## Live-cell Barcoding

Live-cell barcoding is a technique employed at the initial steps of CyTOF analysis. This process involves the labeling of cell surfaces with unique identifiers, followed by pooling and downstream processing that includes both surface and intracellular staining. This streamlined approach allows for the simultaneous analysis of a large number of samples (Figure 1).

The ability of ITCBE to chelate bivalent Pd ions has notably broadened the spectrum of metal isotopes used in CyTOF, catalyzing the development of novel live-cell barcoding techniques that utilize monoisotopic Pd-tagged antibodies. A notable progress in this area was achieved by Mei *et al.*, who devised a strategy using bifunctional ITCBE loaded with Pd isotopes and tagged to CD45, an antigen ubiquitously expressed on hematopoietic cells [31]. Employing a ‘6-choose-3’ barcoding scheme, they successfully achieved the barcoding and pooling of 20 experimental conditions using this method.

However, despite these innovations, ITCBE has demonstrated poor solubility in buffers conducive to effective conjugation, posing significant challenges and limiting its practical application in labeling antibodies with Pd isotopes. This shortcoming necessitated the exploration of alternative chelators, culminating in the utilization of mDOTA [15] for live-cell barcoding. mDOTA is distinguished by its excellent solubility in water and its capacity to chelate both bi- and trivalent metal ions, thereby enabling more efficient antibody conjugation with Pd isotopes and presenting a substantial improvement over ITCBE for live-cell barcoding applications. Live-cell barcoding offers several distinct advantages [42–44]: it provides more streamlined experiments and minimizes batch effects through simultaneous processing of pooled samples. This method reduces technical errors and enhances data interpretation, leading to more precise biological insights. Furthermore, live-cell barcoding conserves valuable samples and is cost-effective by reducing the number of antibodies and reagents needed, and facilitates high-throughput analysis, thereby boosting the potential for new discoveries.

### Optimized live-cell barcoding approach

Traditional live-cell barcoding techniques using ITCBE, BABE and mDOTA, have shown promise for incorporating Pd isotopes into CyTOF applications and various studies have explored the use of these chelators in both intracellular and live-cell barcoding approaches (Table 2). However, these monomeric chelators are limited by their ability to bind only a small number of metal ions, leading to lower signal intensities [31,42]. Additionally, in n-choose-3 barcoding schemes that utilize three CD45 antibodies each tagged with a different metal isotope to barcode a single sample, competitive binding at the same antigenic sites results in diminished signal intensities due to multiple antibodies targeting identical epitopes [31,44]. To address these limitations, we explored the use of MCP9, a polymeric chelator initially developed for conjugating bivalent cadmium (Cd) isotopes [42]. Notably, MCP9 is capable of binding a higher number of bivalent metal ions given its polymeric structure, enhancing signal intensities and the utility of barcoding techniques. Informed by prior developments in chelation chemistry—such as mDTPA, which was initially tailored for lanthanide metals but later found to effectively bind trivalent non-lanthanides like indium (113In and 115In) and bismuth (209Bi) [12,13]—we reasoned that MCP9 could similarly be adapted to chelate bivalent Pd isotopes. Upon confirming that MCP9 can also chelate Pd isotopes we developed a novel barcoding scheme incorporating MCP9 by conjugating it with both Cd and Pd isotopes, utilizing seven Cd isotopes (106Cd, 110Cd, 111Cd, 112Cd, 113Cd, 114Cd, and 116Cd) and three Pd isotopes (104Pd, 105Pd, and 108Pd) to CD45 antibodies [42]. We implemented this approach in a 10-choose-2 combinatorial scheme, leveraging the ubiquitously expressed CD45 antigen on hematopoietic cells, which varies in abundance across different subsets, to validate our barcoding strategy. The selection of CD45 for this proof-of-concept reflects its widespread use in previous studies, underscoring its reliability and effectiveness in validating our barcoding strategy.

#### Advantages:

This strategy allowed us to barcode and pool 45 experimental conditions significantly streamlining our CyTOF experimentations, enhancing our high throughput along with well-established benefits achieved through barcoding.

##### High-throughput:

Opting for two different CD45 antibodies per sample, instead of three, was a strategic decision to maintain optimal signal resolution and ensure higher yields post-sample deconvolution [42]. By constructing each barcode from any two out of ten possible isotopes and pooling, we can analyze numerous samples simultaneously compared to traditional schemes [15,33,42]. This capability is particularly crucial in experiments involving large numbers aimed at generating comprehensive datasets. The strategic use of a combination of Cd and Pd isotopes with MCP9 significantly enhances signal intensity and resolution, which is essential for optimizing sample demultiplexing and achieving higher yields by accurately assigning barcoded cells back to their original identities. Moreover, the flexibility of the 10-choose-2 scheme allows for precise tailoring of experiments to address specific research questions, facilitating the exploration of a broader range of scientific hypotheses.

##### Improving cell yield after sample demultiplexing:

This barcoding strategy also optimizes the use of available resources by reducing the amount of antibodies required, thereby cutting costs and minimizing resource consumption. In CyTOF experiments, where substantial cell loss through multiple processing steps is common—often necessitating 1–2 million cells per sample to ensure adequate counts for analysis—our approach can markedly improve recovery rates. By pooling multiple small samples into larger barcoded groups, we enhance the likelihood of recovering adequate cell numbers for analysis. Additionally, the incorporation of irrelevant or “carrier” cells during the staining process can further improve recovery, particularly in instances of low starting cell numbers [45]. This method proves especially beneficial given that our barcoding scheme allows for the high-throughput processing of numerous features, thus maximizing the effectiveness and efficiency of CyTOF analysis.

##### Enhanced high parametric analysis:

Our barcoding and pooling strategy combines numerous samples into a single mixture, which yields high initial cell counts critical for robust downstream analysis. This mixture can be optionally divided into multiple subsamples, each can be stained with a different panel. Despite this division of pooled samples, it is possible to achieve a cell count of 5,000 to 20,000 cells per each barcoded sample after sample demultiplexing, a range suitable for effective high-dimensional analysis. Achieving this cell number for downstream analysis after demultiplexing is feasible since the initial cell number per sample prior to barcoding typically ranges from 1 to 2 million. Furthermore, the use of sample barcoding significantly reduces cell loss, ensuring the desired cell count per subsample is consistently achieved. This approach not only accommodates the inherent variability in sample heterogeneity with regards to subset composition but also ensures that sufficient cell numbers are maintained across various experimental conditions. By doing so, we enable comprehensive and reliable high-dimensional profiling, maximizing the potential for detailed biological insights. Notably, we can further optimize this process and design panels in a way to share common surface markers, which act as anchors for panel integration. Expressions of markers not directly measured in certain panels can be inferred and imputed using computational approaches, enabling a comprehensive assessment of cellular features. This integration streamlines workflows, maximizes data extraction from each cell, and broadens the scope of proteomic profiling.

### Future directions

In our barcoding approach several strategic approaches can be utilized to increase barcoding depth. In our barcoding scheme, we have not used the rare and more expensive 102Pd isotope. Incorporating this isotope along with 89Y could facilitate a 12-choose-2 scheme, enabling more in-depth demultiplexing. Furthermore, universal barcoding approaches can be developed by integrating antibodies against commonly expressed antigens such as B2M, CD298, and HLA-ABCs. This method allows for the customization of barcoding, where antibodies can be contextually replaced depending on specific analysis needs. This adaptation would improve the versatility and efficiency of our barcoding techniques, aligning our methods with the latest advancements in the field and broadening the scope for future cellular analysis.

## Figures and Tables

**Table 1.** Comparison of Live-cell Barcoding and Intracellular Barcoding Techniques. This table compares two barcoding methods used in CyTOF, focusing on their key features and applications.

Feature	Live-cell Barcoding	Intracellular Barcoding
Defrnition	Barcoding technique that labels cell surfaces with unique identifiers, either metal-tagged antibodies or metal compounds prior to pooling and downstream processing.	Barcoding technique that labels cells with unique mass-tag cell barcoding labels after fixation and permeabilization.
Mass-tag Cell Barcodes (MCBs)	Utilizes metal-tagged antibodies, RuO_4_, OsO_4_ and monoisotopic cisplatin and TeMal compounds.	Monoisotopic TeMal and cisplatin compounds, RuO_4_, OsO_4_ and bifunctional chelators loaded with Pd, In, rhodium or lanthanides.
Chelators	mDOTA, ITCBE, mDTPA and MCP9 are used for generation of metal-tagged antibodies.	mDOTA, ITCBE and BABE are used for generation of meta-loaded MCBs.
Targeted Groups	Targets specific abundant antigens such as CD45, beta- 2-microglobulin, HLA-ABC, CD298, CD29, and CD98, typically using species-specific antibodies.	Labels cellular components that contain amine or sulfhydryl groups, as well as fatty acids and aromatic amino acids.
Cell Type	Requires species-specific antibodies tailored to unique antigens present on cells of a specific species.	Non-species-specific; employs universal tags that indiscriminately bind to generic cellular components.
Viability	Critical; cells must remain viable through the process to ensure accurate subsequent downstream analysis.	Not applicable since cells are already fixed.
Feasibility	Less complex since it avoids the extensive cell preparation steps required for intracellular barcoding.	More complex since it requires extensive cell preparation steps prior to barcoding.
Advantages	Suitable for samples with low cell counts; allows streamlined downstream analysis and facilitates multiplexed analysis through the use of multiple panels.	ideal for detailed studies of intracellular molecules; allows the generation of a greater number of unique combinations as “k” increases beyond two.
Disadvantages	Potential alteration of cell behavior due to binding to target molecules; reduced signal intensity and yield due to competition among MCBs targeting the same antigenic epitopes; requires abundantly expressed antigens for effective barcoding.	Fixation and permeabilization processes can modify antigenic epitopes, resulting in diminished or entirely lost antibody binding capabilities; cell loss due to complex nature of the procedure and numerous wash steps.

**Abbreviations:** ITCBE: isothiocyanobenzyl-EDTA; mDOTA: Maleimido-mono-amide-DOTA; BABE: Bromoacetamidobenzyl-EDTA; MCP9: Maleimido-Cyclohexyl-Phenyl-9; TeMal: Tellurium Maleimide; RuO_4_: Ruthenium Tetroxide; OsO_4_: Osmium Tetroxide; Pd: Palladium; in: indium.

**Table 2.** Key CyTOF studies that have developed and employed various barcoding chemistries and isotopes to enhance cytometric analyses. The table highlights the diversity of approaches tailored to different scientific needs and biological specimens.

Study	Type	Purpose	Chelator/Compound	Isotope	Targeted Groups	Species
Zunder *et al*. [33]	Intracellular	Novel “6-choose-3” barcoding scheme	ITCBE	Pd	Amine groups	Any
Bodenmiller *et al*. [7]	Intracellular	High-throughput CyTOF method	mDOTA	Lanthanides	free sulfhydryl groups	Any
Sumatoh *et al*. [37]	Intracellular	Better chelator	BABE	Pd, In	free sulfhydryl groups	Any
Hartmann *et al*. [44]	Live	Universal live-cell barcoding	N/A	Pt	B2M, CD298	Human
Muftuoglu *et al*. [42]	Live	Extended live-cell barcoding	MCP9	Cd, Pd	CD45	Human
Mei *et al*. [31]	Live	First live-cell barcoding	ITCBE	Pd, In	CD45	Human
Lai *et al*. [38]	Live	Lanthanide-based live-cell Barcoding	DN3	Lanthanides	CD45	Human
Charmsaz *et al*. [43]	Live	Murine live-cell Barcoding	MCP9, X8	Cd, In	CD29, CD98, CD45	Mouse
McCarthy *et al*. [29]	Intracellular, Live	monoisotopic cisplatin-based	Cisplatin	Pt	free sulfhydryl groups	Any
Catena *et al*. [30]	Intracellular, Live	Enhanced multiplexing	RuO4 OsO4	Ru, Os	Fatty acids and aromatic amino acids	Any
Willis *et al*. [32]	Intracellular, Live	Tellurium-based	TeMal	Te	free sulfhydryl groups	Any

**Abbreviations:** ITCBE: Isothiocyanobenzyl-EDTA; mDOTA: Maleimido-mono-amide-DOTA; BABE: Bromoacetamidobenzyl-EDTA; MCP9: Maleimido-Cyclohexyl-Phenyl-9; DN3: A proprietary polymer used in CyTOF; X8: A proprietary polymer used in CyTOF; TeMal: Tellurium Maleimide; RuO_4_: Ruthenium Tetroxide; OsO_4_: Osmium Tetroxide; Cd: Cadmium; Pd: Palladium; In: Indium; Te: Tellurium; Ru: Ruthenium; Os: Osmium.
